# Chemsex as a challenge for emergency medical teams: a narrative review

**DOI:** 10.3389/fpubh.2026.1921570

**Published:** 2026-10-26

**Authors:** Michał Czapla, Krzysztof Griesmann, Raúl Juárez-Vela, Antonio Martinez-Sabater, Christopher S. Lee, Piotr Karniej, Łukasz Pietrzykowski, Aleksander Mickiewicz, Kamil Kędzierski, Jakub Wojciechowski, Andrzej Raczyński, Jacek Smereka

**Affiliations:** 1 Department of Emergency Medical Service, Faculty of Nursing and Midwifery, Wroclaw Medical University, Wroclaw, Poland; 2 Group for Research in Healthcare (GRUPAC), Faculty of Health Science, University of La Rioja, Logroño, Spain; 3 Institute of Neurosciences of Castile and León (INCYL), University of Salamanca, Biomedical Research Institute of Salamanca (IBSAL), Valencia, Spain; 4 Faculty of Nursing and Podology, Nursing Care and Education Research Group (GRIECE), University of Valencia, Valencia, Spain; 5 Care Research Group (INCLIVA), Hospital Clínico Universitario de Valencia, Valencia, Spain; 6 William F. Connell School of Nursing, Boston College, Boston, MA, United States; 7 Faculty of Finances and Management, WSB MERITO University in Wrocław, Poland; 8 Department of Anaesthesiology and Intensive Care, Antoni Jurasz University Hospital No. 1 in Bydgoszcz, Bydgoszcz, Poland

**Keywords:** chemsex, emergency medical services, harm reduction, polysubstance use, prehospital care

## Abstract

Chemsex, defined as the intentional use of psychoactive substances before or during sexual activity, has emerged as an important public health concern and an increasingly relevant challenge for emergency medical services (EMS). Although most frequently described among men who have sex with men, sexualized substance use is increasingly reported among heterosexual men and women, indicating a broader at-risk population. Commonly used substances include methamphetamine, mephedrone, gamma-hydroxybutyrate/gamma-butyrolactone (GHB/GBL), alkyl nitrites, and other psychoactive agents, frequently used in combination during prolonged sexual sessions. Such practices are associated with a wide spectrum of acute complications, including agitation, psychosis, hyperthermia, cardiovascular instability, respiratory depression, altered consciousness, and mixed toxidromic presentations. This narrative review examines current evidence regarding chemsex-associated emergencies from a prehospital and emergency medicine perspective. Particular attention is given to syndromic clinical presentations, including hyperstimulatory syndromes, behavioral disturbances, acute GHB/GBL toxicity, and polysubstance-related emergencies. Operational challenges relevant to EMS teams, including scene safety, communication barriers, stigma, occupational risks associated with slamsex, and management of agitated patients, are also discussed. Chemsex-related emergencies share many clinical features with other substance-related emergencies. However, recognition of the sexualized substance use context may provide additional information relevant to prehospital assessment, including intentional drug combinations, repeated dosing, potential drug interactions, communication barriers, and occupational safety considerations. A syndromic approach focused on recognition of dominant clinical patterns rather than identification of individual substances may therefore provide the most practical framework for prehospital assessment and management. Although chemsex-associated emergencies are documented, evidence regarding their frequency and trends remains limited, particularly in prehospital settings. Further research is needed to support the development of evidence-based prehospital protocols, educational initiatives, and integrated care pathways.

## 1 Introduction

Chemsex has emerged as a complex public health and emergency care challenge involving the intentional use of psychoactive substances before or during sexual activity to facilitate, intensify, prolong, or disinhibit sexual experiences. Although most commonly described among men who have sex with men (MSM), sexualized substance use has also been reported among heterosexual men and women, indicating that chemsex-related harms may extend beyond populations traditionally associated with the practice (1, 2). In the absence of a universally accepted definition, chemsex is generally associated with substances such as methamphetamine, mephedrone, and gamma-hydroxybutyrate/gamma-butyrolactone (GHB/GBL) (3, 4). Importantly, these substances are not specific to chemsex and are also used in other recreational contexts. Within chemsex settings, however, substances may be intentionally selected for their specific or complementary effects on sexual experience, including stimulation, disinhibition, enhanced sensory perception, and relaxation (5). Chemsex is associated with prolonged sexual sessions, multiple sexual partners, condomless intercourse, and frequent polysubstance use, contributing to increased risks of infectious, psychiatric, and toxicological complications (5). Reported prevalence among MSM populations in Europe varies substantially according to study methodology and operational definitions although recent meta-analytic estimates suggest that approximately 16% (95% CI: 11–21%) of MSM engage in chemsex practices (4). Chemsex-associated substance use has additionally been linked to sexually transmitted infections, adverse mental health outcomes, and severe acute intoxications requiring emergency medical intervention (6, 7). Importantly, chemsex-related intoxications may differ clinically from conventional recreational drug intoxications because of repeated dosing, prolonged exposure, polysubstance use, and delayed healthcare presentation (7).

European surveillance data document acute toxicity associated with substances also used in chemsex, although these presentations cannot necessarily be classified as chemsex-related (8). Local observational studies have reported increasing emergency department presentations: a Barcelona study documented an increase in chemsex-related intoxications among people living with HIV between 2018 and 2020 (7), whereas a study from central London reported increasing presentations involving methamphetamine toxicity between 2005 and 2018, without establishing a chemsex context for all cases (9). These findings demonstrate local trends but do not establish an overall increase in chemsex-related emergencies across Europe. Importantly, identification of the chemsex context remains challenging because the substances involved are not specific to chemsex and, in the absence of a universally accepted definition, definitions and included substances vary considerably across studies (2, 10, 11). An additional challenge in interpreting these trends is the possible underrecognition and underdocumentation of the chemsex context in emergency care. Patients may be recorded as presenting with acute drug intoxication, altered consciousness, or behavioral disturbances without documentation of the sexual context of substance use. Conversely, increased clinical awareness and more systematic identification of chemsex may contribute to an apparent rise in documented cases, independently of changes in the actual incidence. Consequently, observed trends should be interpreted cautiously, as they may reflect changes in recognition and documentation as well as changes in the underlying occurrence of chemsex-related emergencies. Accordingly, classification of an emergency presentation as chemsex-related requires information on the sexual context and intentionality of substance use rather than identification of the substance alone (7, 11). Polydrug exposure is common in acute recreational drug toxicity more broadly and is therefore not specific to chemsex-related emergencies (12). Patients may present with fluctuating consciousness, psychomotor agitation, respiratory compromise, hyperthermia, or cardiovascular instability suggesting polydrug exposure. In many cases, substance histories are incomplete or unreliable, while unknown drug composition further complicates prehospital assessment (13). Chemsex-associated emergencies may also create substantial operational challenges for emergency medical services (EMS) teams. For example, GHB-related toxicity is frequently characterized by abrupt fluctuations between deep sedation, agitation, respiratory compromise, and altered consciousness (13). Moreover, stimulant-associated toxicity may additionally contribute to arrhythmias, seizures, rhabdomyolysis, and cerebrovascular complications, particularly during prolonged sessions involving sleep deprivation, dehydration, and repeated dosing (14).

Despite the rapidly expanding literature on chemsex, there is limited evidence to support EMS and prehospital care. Most published studies focus predominantly on HIV prevention, sexually transmitted infections, addiction medicine, psychiatric comorbidities, and psychosocial determinants of chemsex participation rather than acute emergency management (15). At the same time, chemsex-related emergencies may involve severe toxicological and behavioral presentations, including psychomotor agitation, psychosis, hyperthermia, seizures, cardiovascular instability, respiratory depression, and polysubstance intoxication requiring urgent medical intervention (11, 14). Current literature provides limited practical guidance for paramedics and other prehospital staff regarding scene safety, recognition of chemsex-associated toxidromes, sedation strategies, airway management, or transportation decisions. Furthermore, there are no standardized EMS protocols specifically addressing chemsex-related emergencies, despite the clinical and operational challenges associated with these presentations (11). The absence of evidence-based prehospital guidance may contribute to delayed recognition of life-threatening complications, inconsistent clinical management, and increased operational risks for EMS teams. Previous work, particularly that of Kingsley (11), has provided an important practice-oriented introduction to chemsex for EMS clinicians, highlighting relevant substances, clinical presentations, and the importance of non-judgemental care. Building on this foundation, the present review synthesizes clinical and toxicological evidence within a syndromic framework, with particular emphasis on overlapping presentations, prehospital assessment and management priorities, and limitations of the available EMS-specific evidence. Rather than proposing a distinct treatment algorithm for chemsex-related emergencies, this review examines how recognition of the chemsex context may inform the application of established emergency care principles.

This narrative review critically examines the current evidence regarding chemsex-associated emergencies from a prehospital and EMS perspective. Particular emphasis is placed on toxidromic presentations, operational challenges, scene safety, clinical management, and implications for paramedic practice and EMS systems. The review additionally identifies current gaps in prehospital management and priorities for future clinical protocols and research.

## 2 Materials and methods

A narrative review was conducted to examine the clinical, operational, and toxicological challenges associated with chemsex-related emergencies in prehospital and emergency care settings. Relevant literature was identified through searches of MEDLINE (via PubMed), EMBASE, and the Cochrane Central Register of Controlled Trials (CENTRAL). The initial literature search was conducted between March and June 2026, with relevant publications subsequently added during manuscript preparation and revision. Search terms included combinations of Medical Subject Headings and free-text keywords related to chemsex, sexualized drug use, GHB, GBL, methamphetamine, mephedrone, synthetic cathinones, poppers, emergency medical services, prehospital care, acute intoxication, overdose, agitation, and harm reduction. Sources were considered for inclusion if they addressed chemsex-related substance use, acute toxicity, pharmacological interactions, emergency presentations, clinical management, or operational challenges with potential relevance to prehospital care. Eligible sources included peer-reviewed studies, clinical guidelines, systematic and narrative reviews, observational studies, case reports, and selected expert commentaries. Evidence from emergency departments, hospital-based studies, toxicological investigations, and experimental research was also considered when its findings could inform prehospital assessment or management. English-language publications were considered. Literature identification and selection were undertaken by the authors according to their respective contributions to the thematic sections of the review, with sources selected on the basis of clinical relevance and applicability to emergency and prehospital care. Sources without a meaningful connection to acute clinical presentations, toxicological risks, patient management, or operational aspects relevant to emergency and prehospital care were generally excluded. Preference was given to contemporary literature addressing toxidromic presentations, prehospital management, operational challenges, and implications for emergency medical services. Selected grey literature, including institutional reports and community-based publications, was also obtained from official organizational websites to provide relevant epidemiological and public health context (8, 16). Given the heterogeneity of the available literature and the limited EMS-specific evidence, a narrative review methodology was considered the most appropriate approach to synthesize current knowledge and identify implications for prehospital care. As this narrative review does not aim to develop or grade clinical recommendations, the supporting evidence is discussed with reference to its source, including clinical guidelines, observational studies, and expert consensus, while acknowledging the limited availability of EMS-specific data.

## 3 Chemsex-associated emergencies: a syndromic approach

### 3.1 Understanding chemsex-associated emergencies in prehospital care

Chemsex-associated emergencies represent an increasingly relevant challenge for EMS, particularly in large urban settings and among populations engaging in sexualized drug use. Although methamphetamine, mephedrone, GHB/GBL, ketamine, and cocaine remain the substances most commonly associated with chemsex, clinical presentations rarely reflect isolated single-substance intoxication. Instead, EMS teams frequently encounter complex patterns of polysubstance exposure accompanied by rapidly evolving neuropsychiatric, cardiovascular, and toxicological manifestations (11, 14, 15). Contemporary chemsex practices frequently involve prolonged sessions, repeated dosing, co-administration of stimulants, sedatives, and alkyl nitrites (“poppers”), as well as intravenous administration of psychoactive substances in a sexual context(“slamsex”), increasing the risk of severe toxicity, psychosis, respiratory compromise, traumatic injury, and fluctuating consciousness (11, 17–19).

From a prehospital perspective, chemsex-associated emergencies are particularly difficult to assess because patient histories are often incomplete, delayed, unreliable, or entirely unavailable. Participants may be unconscious, severely intoxicated, psychotic, or reluctant to disclose substance use because of stigma, criminalization, or fear of discrimination (11, 16). Familiarity with community-specific terminology may provide important contextual clues during EMS encounters, particularly when patients, partners, or bystanders use colloquial rather than pharmacological terms. Several commonly used expressions may indicate specific substances, routes of administration, or substance-related effects that are not immediately apparent during routine history taking. Selected terms relevant to prehospital care are summarized in Table 1.

**Table 1:** Common chemsex terminology and its clinical relevance for EMS teams.*

Term	Meaning	Clinical relevance for EMS
Chemsex	Intentional use of psychoactive substances before or during sexual activity	Suggests prolonged exposure, repeated dosing, polysubstance use, delayed presentation, and contextual risks beyond “standard intoxication”
Tina/T/crystal	Methamphetamine	May indicate sympathomimetic toxicity: agitation, paranoia, psychosis, hyperthermia, tachycardia, hypertension, seizures, rhabdomyolysis
Mephedrone/4-MMC/Meow Meow	Synthetic cathinone with stimulant effects	May indicate sympathomimetic toxicity, including agitation, tachycardia, hypertension, hyperthermia, and possible cardiovascular complications
G	GHB/GBL	May indicate sedative-hypnotic toxicity: coma-like state, respiratory depression, loss of airway reflexes, aspiration risk, abrupt awakening, fluctuating consciousness
PnP/party and play	Sexual activity combined with psychoactive substance use	Helps identify chemsex context, possible hidden polysubstance exposure, prolonged session, and multiple intoxicated individuals
Slamsex/slamming	Intravenous drug use during sex	Higher risk of rapid-onset toxicity, overdose, sepsis, abscesses, needle-stick hazards, and blood-borne virus exposure
Booty bumping	Rectal administration of drugs	May cause faster absorption and unpredictable toxicity; relevant when oral/nasal/intravenous use is denied
Crystal dick	Methamphetamine-associated erectile dysfunction resulting from sympathomimetic and vasoconstrictive effects	May indicate recent stimulant exposure and possible concurrent use of PDE5 inhibitors.
Poppers	Volatile alkyl nitrite inhalants (e.g., amyl nitrite, butyl nitrite, isopropyl nitrite) used to intensify sexual experiences and facilitate sexual activity.	Risk of acute hypotension, syncope, reflex tachycardia, methemoglobinemia, and visual symptoms; potentially severe hypotension when combined with PDE5 inhibitors
Viagra/Cialis/erectile pills	PDE5 inhibitors, including sildenafil, tadalafil, vardenafil, or avanafil	Important co-exposure clue; combination with poppers/nitrates may precipitate profound hypotension, collapse, or shock
G chart/dosing timer	Written or phone-based record used to time repeated GHB/GBL doses	Suggests repeated dosing and risk of cumulative toxicity, recurrent sedation, respiratory compromise, or abrupt clinical deterioration
K/Special K	Ketamine	May indicate dissociative toxicity, including altered consciousness, agitation, hallucinations, impaired coordination, and cardiovascular effects; co-use with other depressants may increase the risk of respiratory compromise.

^*^Adapted from selected chemsex literature (1, 4, 11, 58, 68–70).

Furthermore, novel psychoactive substances (NPS) used in chemsex include synthetic cathinones, particularly mephedrone (4-MMC), as well as other emerging synthetic stimulants. The composition and potency of these substances may be uncertain, further complicating the prediction of clinical effects and prehospital assessment (8, 20, 21). Overlapping toxidromes are therefore common, with stimulant toxicity coexisting alongside sedative effects, fluctuating consciousness, hyperthermia, psychomotor agitation, seizures, or cardiorespiratory compromise (14, 22).

Importantly, chemsex-related presentations are highly dynamic. Fluctuating clinical states may occur following repeated dosing and mixed exposure to stimulants and depressants, particularly methamphetamine and GHB/GBL, resulting in rapidly evolving presentations that complicate prehospital assessment (14, 16). This syndromic instability limits the usefulness of substance-specific diagnostic frameworks during early emergency assessment. As in acute recreational drug toxicity more broadly, EMS teams managing chemsex-related emergencies frequently need to initiate treatment before toxicological confirmation is available and without definitive identification of the substances involved (12). Moreover, the presenting signs and symptoms are often non-specific and may overlap with primary psychiatric disorders or acute medical and neurological conditions unrelated to substance use, further complicating prehospital differential diagnosis (14). Consequently, early recognition of dominant toxidromic patterns including sympathomimetic, sedative-hypnotic, serotonergic, or mixed presentations, may provide a more clinically useful approach than attempting to identify a single causative drug (23). Therefore, chemsex-associated emergencies should be conceptualized primarily as overlapping clinical syndromes rather than isolated intoxications. For EMS teams, a syndromic framework may better support rapid assessment, risk stratification, early stabilization, and safer prehospital management in this increasingly complex patient population. Figure 1 illustrates a syndromic approach to chemsex-associated emergencies, highlighting the progression from risk factors and polysubstance exposure to dominant clinical presentations and EMS management priorities.

**Figure 1:**
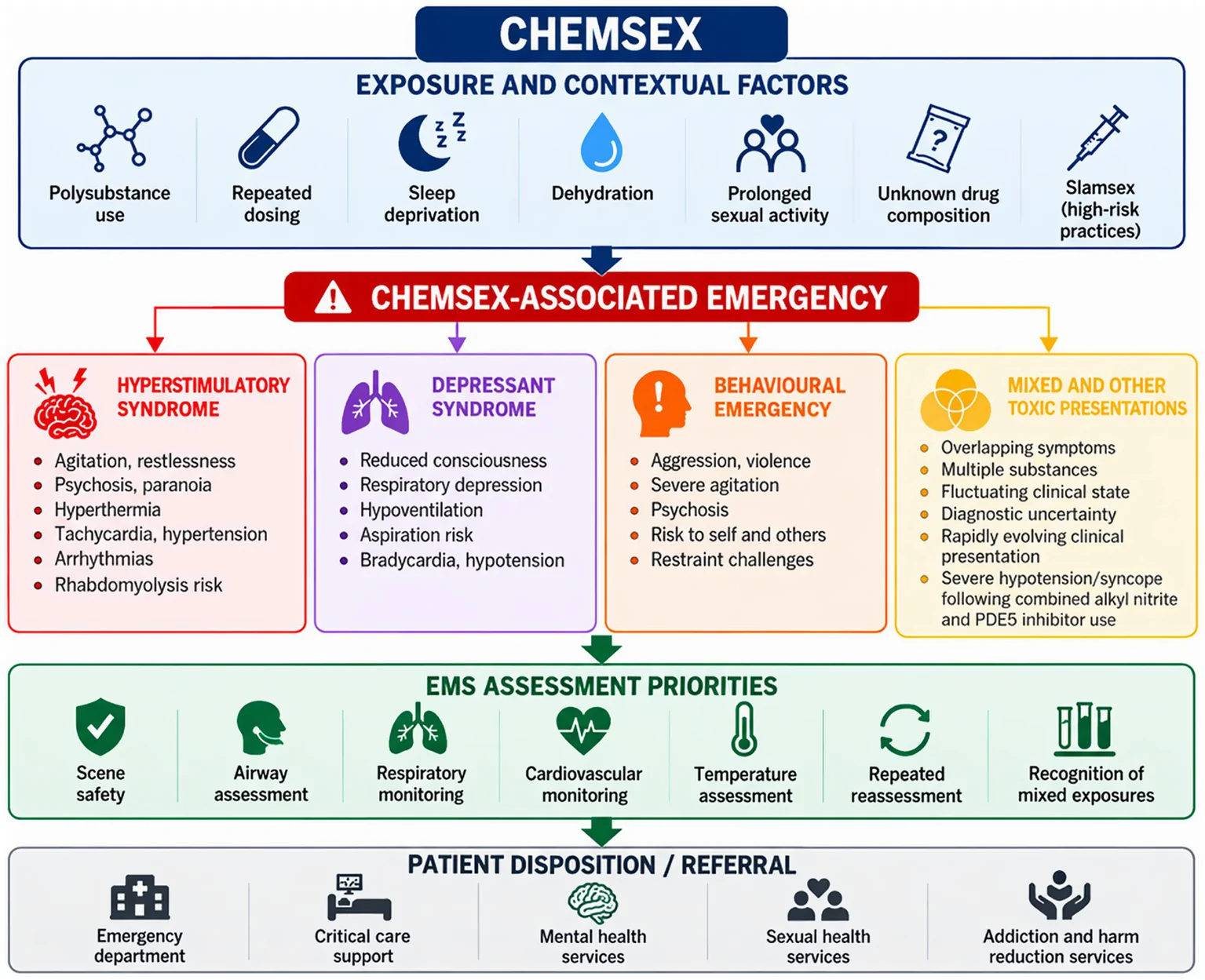
Syndromic approach to chemsex-associated emergencies in EMS practice. Chemsex-related emergencies are frequently characterized by polysubstance exposure, repeated dosing, prolonged wakefulness, dehydration, and rapidly evolving clinical manifestations. Recognition of the dominant clinical syndrome may provide a more practical framework for prehospital assessment and management than identification of individual substances alone.

### 3.2 Clinical presentations and prehospital management of chemsex-associated emergencies

Chemsex-associated emergencies encompass a broad spectrum of clinical presentations driven not only by psychoactive substance use but also by prolonged exposure, repeated dosing, sleep deprivation, dehydration, and sustained physical exertion. Although polysubstance use is common in substance-related emergencies more broadly, chemsex may involve intentional and functionally selected combinations of psychoactive substances, often used sequentially or repeatedly during prolonged sexual sessions to achieve complementary effects (5, 19). This pattern of exposure may result in overlapping and rapidly evolving clinical manifestations, complicating identification of the substances involved during the initial prehospital assessment (14, 24). Hyperstimulatory presentations associated with methamphetamine, mephedrone, cocaine, or MDMA may manifest with agitation, psychosis, hyperthermia, and cardiovascular instability (14, 24, 25). Ketamine-related toxicity may present as dissociation, perceptual disturbances, impaired situational awareness, agitation, hypertension, and tachycardia. When combined with stimulants or other psychoactive substances, clinical assessment may be further complicated by overlapping toxidromic features (26, 27). In contrast, GHB/GBL-related toxicity typically presents with fluctuating consciousness, respiratory depression, and sudden transitions between deep sedation and agitation (24, 28). Mixed clinical presentations may occur when multiple psychoactive substances are used during a chemsex session, potentially resulting in overlapping neurological, respiratory, and cardiovascular effects (5, 19). Consequently, a syndromic approach may provide a more practical framework for EMS teams than a substance-specific model. The following sections summarize the principal clinical syndromes encountered during chemsex-associated emergencies and discuss their relevance for prehospital assessment and management.

#### 3.2.1 Hyperstimulatory syndrome, agitation, and behavioral disturbance

Hyperstimulatory presentations are among the most frequently encountered and potentially life-threatening chemsex-associated emergencies. Hyperstimulatory presentations are most commonly associated with methamphetamine, mephedrone and other synthetic cathinones, cocaine, and MDMA, either alone or in combination (14, 24, 29). In contrast to isolated recreational drug use, chemsex-related stimulant toxicity is often compounded by repeated dosing, prolonged wakefulness, dehydration, physical exertion, and concurrent use of multiple psychoactive substances (24, 30).

The clinical picture is dominated by a sympathomimetic toxidrome characterized by psychomotor agitation, anxiety, paranoia, aggression, insomnia, tachycardia, hypertension, diaphoresis, mydriasis, and hyperthermia (14, 24, 29). Patients may exhibit severe behavioral disturbance, impaired judgement, impulsivity, and reduced situational awareness, creating substantial challenges for scene safety and prehospital assessment (22, 24). Acute psychotic symptoms, including hallucinations, persecutory delusions, and violent behavior, are well-recognized complications of stimulant exposure and frequently necessitate emergency intervention (22, 24).

Hyperthermia represents a particularly important red flag for EMS teams. Sustained sympathetic activation, excessive muscular activity, and impaired heat dissipation may lead to rapidly rising core body temperature and a heat-stroke-like syndrome associated with coagulopathy, rhabdomyolysis, acute kidney injury, and multiorgan dysfunction (30, 31). The risk of severe toxicity is further increased by prolonged chemsex sessions, during which dehydration, sleep deprivation, and repeated stimulant administration are common (24, 30).

Cardiovascular complications represent one of the most important causes of severe morbidity and mortality among individuals exposed to stimulant-associated chemsex substances (32, 33). Excessive catecholaminergic stimulation may precipitate cardiac arrhythmias, myocardial ischemia, acute coronary syndromes, cerebrovascular events, and sudden cardiac arrest (24, 32, 34). Hyperthermia, electrolyte disturbances, and volume depletion may further exacerbate cardiovascular instability and increase the risk of rapid clinical deterioration (24, 30).

For EMS teams, the combination of severe agitation, hyperthermia, cardiovascular instability, and altered mental status should prompt immediate recognition of a potentially life-threatening hyperstimulatory syndrome. Early attention to scene safety, physiological monitoring, rhythm management, active cooling, fluid resuscitation, and timely transport is essential to reduce the risk of catastrophic complications (24, 30). Active cooling should be initiated promptly when clinically significant stimulant-associated hyperthermia is suspected, particularly in patients with altered mental status. Although temperatures above approximately 40.5 °C indicate severe hyperthermia and may require rapid cooling methods, treatment should not be delayed until this threshold is reached (35).

Psychomotor agitation is one of the most common clinical manifestations of acute stimulant toxicity and represents a frequent reason for EMS activation. Excessive dopaminergic and noradrenergic stimulation leads to behavioral dysregulation, aggression, impulsivity, and impaired judgment, often creating safety risks for patients, bystanders, and healthcare providers (14, 24). Methamphetamine-associated agitation may range from mild restlessness to severe behavioral emergencies requiring pharmacological sedation or physical restraint. Acute presentations may be accompanied by paranoia, aggression, and psychotic symptoms, which can complicate emergency assessment and management (22, 24).

From an EMS perspective, agitated patients represent a significant operational challenge. Safety concerns frequently necessitate restraint procedures or pharmacological sedation when de-escalation strategies fail. Qualitative research involving EMS clinicians managing agitation in older adults has highlighted the ethical and operational challenges of balancing patient autonomy, provider safety, and the potential harms of restraint (36). Large-scale prehospital data indicate that chemical sedation is an established component of behavioral emergency management, although it is required in only a minority of cases. Although serious adverse events following sedation are relatively uncommon, continuous monitoring remains necessary because respiratory compromise, oxygen desaturation, and airway interventions may occur after sedative administration (37).

#### 3.2.2 Depressant presentations: acute GHB/GBL intoxication

Depressant presentations constitute a major proportion of severe chemsex-associated emergencies and are most commonly linked to gamma-hydroxybutyrate (GHB), gamma-butyrolactone (GBL), alcohol, benzodiazepines, opioids, or combinations thereof. In contrast to hyperstimulatory syndromes, these presentations are characterized predominantly by central nervous system and respiratory depression, often requiring urgent airway assessment and supportive care (13, 38, 39). A population-based analysis from New South Wales, Australia, identified 9,612 GHB-related emergency department presentations between July 2015 and January 2024, of which 79.0% arrived by ambulance (40). Profound impairment of consciousness is the hallmark feature of severe GHB toxicity. Emergency department studies consistently report markedly reduced Glasgow Coma Scale (GCS) scores, with many patients presenting unconscious or unresponsive (13, 38). In a Swiss cohort, coma occurred in approximately two-thirds of cases, while Australian studies similarly identified severe reductions in consciousness as the dominant clinical manifestation of acute GHB intoxication (13, 39). Respiratory depression, hypoventilation, loss of protective airway reflexes, bradycardia, and hypotonia are common accompanying features and represent the principal causes of life-threatening deterioration (40, 41). A characteristic feature distinguishing GHB from many other depressant agents is its short duration of action. Patients may transition rapidly from profound unconsciousness to spontaneous awakening, often within a relatively short period following presentation (28). However, transient clinical improvement should not be interpreted as resolution of toxicity, as recurrent sedation may occur, particularly following repeated dosing or co-ingestion of other depressant substances (28, 39). In a retrospective emergency department study, the median time to regain consciousness following GHB intoxication was 90 min (range: 3–400 min) (39). However, this finding does not establish a safe observation period or discharge threshold for prehospital care. Patients who regain consciousness should undergo reassessment of their neurological and respiratory status, with particular attention to possible co-ingestion and recurrent sedation. When a patient declines transport, decision-making capacity should also be assessed in accordance with applicable clinical and legal procedures.

Polydrug exposure is common among patients presenting with GHB-related toxicity and may substantially modify clinical presentation. Contemporary studies demonstrate frequent co-use of alcohol, methamphetamine, benzodiazepines, and opioids (13, 40, 41). Alcohol co-ingestion warrants particular attention because a controlled study in 16 healthy volunteers found that combined GHB and ethanol exposure caused a modest but statistically significant additional reduction in oxygen saturation (maximum mean decrease of 2.1 percentage points), alongside more vomiting and episodes of transient hypotension (42). Postmortem toxicological studies of GHB-related deaths frequently identify co-exposure to other central nervous system depressants, particularly opioids and sedative-hypnotic agents (41, 43). When opioid co-ingestion is suspected in patients with respiratory depression, naloxone should be considered in accordance with established emergency care protocols. However, naloxone does not reverse the direct effects of GHB/GBL, and airway support and adequate ventilation remain essential (44, 45). For EMS teams, airway and respiratory management remain the highest priorities. Depressed consciousness should prompt immediate assessment of airway patency, respiratory rate, oxygenation, and adequacy of ventilation. Vomiting is common and, when combined with loss of airway reflexes, creates a significant risk of aspiration. Consequently, airway positioning, suction readiness, supplemental oxygen, and continuous monitoring are essential components of prehospital management (40, 41). Patients presenting with severe reductions in consciousness, respiratory compromise, suspected mixed intoxication, or recurrent sedation should be transported urgently for ongoing observation and advanced supportive care. A practical clinical pearl for EMS teams is that profound coma accompanied by subsequent rapid spontaneous recovery should raise suspicion of GHB/GBL toxicity, particularly in the context of suspected chemsex-related polysubstance use (28, 39). In patients with suspected GHB/GBL dependence, EMS clinicians should also consider the possibility of withdrawal, which may begin within 1–6 h after the last dose and progress to severe agitation, autonomic instability, or delirium (46). Information regarding frequent GHB/GBL use, the time of the last dose, and suspected withdrawal should be communicated during hospital handover.

#### 3.2.3 Mixed and polysubstance presentations

Recent conceptual work suggests that drug combinations during chemsex are often intentional and functional rather than random. Substances may be selected for complementary effects, including stimulation, disinhibition, emotional connectedness, altered sensory perception, dissociation, or relaxation (5). The Pharmacosex Wheel framework is useful clinically because it explains why EMS teams may encounter overlapping sympathomimetic, sedative, dissociative, and vasodilatory effects in the same patient (5). In practice, this means that agitation, paranoia, hyperthermia, respiratory depression, hypotension, syncope, and fluctuating consciousness may coexist or evolve rapidly during a single prehospital encounter (5).

GHB/GBL is particularly relevant in this context because of its potential for acute toxicity and its co-use with other psychoactive substances during chemsex (19). Emergency department and ambulance-based studies consistently show that GHB-related toxicity is commonly associated with severe reductions in consciousness, respiratory depression, and need for airway support (13, 28, 38, 39). In Australian cohorts, co-ingestion was frequent, with methamphetamine and alcohol among the most commonly identified substances (13, 38). This is relevant for EMS because stimulant co-use may obscure early sedation, whereas alcohol, benzodiazepines, and opioids may potentiate respiratory depression and increase aspiration risk (28, 38, 39). The interaction between GHB and alcohol is clinically relevant, although the available evidence requires cautious interpretation. In a controlled study involving 16 healthy volunteers receiving therapeutic-dose GHB, co-administration with ethanol produced a maximum mean reduction in oxygen saturation of approximately 2.1 percentage points and was associated with more vomiting and transient hypotension; however, the combination did not lower systolic blood pressure more than ethanol alone (42). Furthermore, a retrospective study of 78 GHB-related intoxications found no statistically significant association between alcohol co-use and coma (77% vs. 62%; *p* = 0.052) or time to recovery among comatose patients (142 vs. 89 min; *p* = 0.07) (39).

Clinical and mortality data also indicate that fatal GHB-related poisonings often involve other central nervous system depressants, including opioids and sedative-hypnotics (41). Therefore, a patient who appears to have “simple GHB intoxication” should be treated as a potential mixed depressant overdose until serial assessment demonstrates sustained recovery (28, 39, 42).

#### 3.2.4 Other clinically relevant exposures: alkyl nitrites and PDE-5 inhibitors

Alkyl nitrites (‘poppers’) represent a clinically relevant but potentially under-recognized exposure in chemsex-related emergencies. Alkyl nitrites are used to enhance sexual pleasure, produce a short “rush,” and facilitate sexual activity through smooth-muscle relaxation (47). Poppers are also frequently used in chemsex settings to facilitate receptive anal intercourse and are strongly associated with group sex, condomless intercourse, and other high-risk sexual behaviors among MSM populations (48, 49). Population-level data show that popper use is substantially more common among gay men than among heterosexual men, and qualitative work suggests that poppers are often normalized within sexual networks and introduced by sexual partners (18, 47). Although many users perceive poppers as low risk, adverse effects include headache, dizziness, hypotension, retinal toxicity, visual impairment, methemoglobinemia, and severe hypotensive reactions (47, 50). The combination of poppers with phosphodiesterase-5 inhibitors is one of the most important “hidden” interactions for EMS. This combination is frequently reported in contemporary chemsex settings and may substantially increase the risk of profound hypotension and cardiovascular collapse (51, 52). Sildenafil, tadalafil, vardenafil, or other erectile dysfunction medications may be used to counter stimulant-associated erectile dysfunction or to sustain prolonged sexual activity (5, 47). Because both alkyl nitrites and phosphodiesterase-5 inhibitors promote vasodilation, co-use can precipitate profound hypotension, syncope, myocardial ischemia, cardiovascular collapse, and rarely death (47, 50). This potential interaction should be considered in patients presenting with unexplained hypotension, collapse, or syncope during or shortly after sexual activity (53). Importantly, recent use of PDE5 inhibitors also has implications for the management of suspected acute coronary syndromes. EMS clinicians should specifically assess recent PDE5 inhibitor use before administering nitrates, as this combination may cause severe hypotension. According to the 2025 ACC/AHA guidelines, nitrates should be avoided within 12 h of avanafil, 24 h of sildenafil or vardenafil, and 48 h of tadalafil (54). Severe methemoglobinemia should also be considered in patients presenting with cyanosis or unexplained hypoxemia following recent popper use, particularly when oxygen saturation appears disproportionately low relative to the clinical examination (50).

For EMS teams, the practical implication is clear: chemsex-related emergencies should be approached as mixed-exposure events unless there is strong evidence to the contrary. Self-report may be incomplete because of stigma, impaired consciousness, fear of disclosure, uncertain drug composition, or inability to recall repeated dosing during prolonged sessions (5, 19, 40). Routine toxicology screening may also fail to identify novel psychoactive substances or rapidly cleared compounds such as GHB (39, 40). Assessment should therefore prioritize the dominant clinical syndrome, repeated reassessment, airway and respiratory monitoring, temperature measurement, cardiovascular surveillance, and targeted questioning about GHB/GBL, stimulants, alcohol, benzodiazepines, opioids, poppers, and erectile dysfunction medications (28).

## 4 EMS challenges and prehospital management

### 4.1 Management of agitation, aggression, and behavioral emergencies

The prehospital environment presents unique operational constraints compared with hospital-based care. EMS teams are required to perform rapid assessments, identify potentially reversible causes of agitation, and simultaneously maintain scene safety in unpredictable environments. National EMS recommendations emphasise that de-escalation strategies should be prioritized whenever feasible, while physical or pharmacological restraint should be considered only when less restrictive interventions have failed and there is an immediate risk of harm (55). International recommendations similarly support a stepwise approach to acute agitation, with verbal and non-verbal de-escalation whenever feasible, followed by pharmacological treatment when agitation is severe, escalating, or poses an immediate risk to the patient or others. In stimulant-induced agitation, benzodiazepines are recommended as first-line pharmacological treatment, while antipsychotic agents should be considered when prominent psychotic symptoms are present. In severe agitation refractory to benzodiazepines and antipsychotics, ketamine may be considered when rapid behavioral control is required for patient or provider safety. Pharmacological management should be guided by the suspected cause of agitation and accompanied by appropriate cardiorespiratory and physiological monitoring, particularly in patients with suspected polysubstance exposure (35, 56). In patients with suspected polysubstance exposure, pharmacological sedation requires particular caution because concurrent GHB/GBL, alcohol, or other central nervous system depressants may increase the risk of respiratory compromise, particularly as stimulant effects diminish. In a large retrospective EMS cohort, ketamine was associated with higher rates of invasive airway intervention (2.0% vs. 0.4%) and severe oxygen desaturation (2.6% vs. 1.3%) compared with benzodiazepines (37). Although these findings do not establish causality or specifically address chemsex-related emergencies, they highlight the importance of individualized sedation decisions, continuous respiratory monitoring, and readiness for airway intervention.

A recent qualitative study involving EMS clinicians in Canada identified insufficient training, limited organisational support, and complex ethical considerations related to the management of agitation and restraint use in older adults (36). Although these findings are not specific to substance-related emergencies, they illustrate broader operational challenges relevant to prehospital agitation management (36). When physical restraint is unavoidable, positions that restrict ventilation, particularly prone restraint, should be avoided because of the risk of positional asphyxia. Continuous assessment of respiratory and cardiovascular status is essential in restrained patients (55). These challenges may be particularly pronounced during chemsex-related emergencies, where polysubstance use, prolonged wakefulness, dehydration, and psychiatric comorbidities frequently coexist.

### 4.2 Communication barriers, stigma, and disclosure

Effective assessment of chemsex-related emergencies relies heavily on accurate history taking. However, disclosure of sexual behaviors, substance use, HIV status, and participation in chemsex may be hindered by anticipated stigma. Evidence from sexual minority populations living with HIV demonstrates that experiences of discrimination and social exclusion are associated with lower trust in healthcare providers and poorer perceptions of healthcare quality (57). Individuals who engage in chemsex often belong to populations that have historically experienced healthcare-related stigma, including gay, bisexual, and other men who have sex with men (GBMSM). Fear of judgment may contribute to incomplete disclosure regarding substances consumed, routes of administration, timing of drug use, or associated sexual practices (58, 59). Recent evidence from a national cross-sectional study among Polish paramedics suggests that, despite relatively high attitudinal awareness, clinical preparedness for caring for LGBT patients remains low, supporting the need for structured, practice-oriented training in EMS settings (60). In a cross-sectional survey of 1,203 sexual minority men in Spain who had engaged in chemsex, only 4.0% reported seeking emergency care following a chemsex session for reasons other than HIV post-exposure prophylaxis (PEP). However, among participants who had never sought emergency care after chemsex, 21.8% reported having experienced a situation in which they felt such care was needed (61). These findings suggest that emergency healthcare utilization may not fully reflect perceived unmet care needs in this population. These communication challenges may be amplified in emergency and prehospital settings, where limited time, clinical urgency, impaired consciousness, agitation, and the need for rapid decision-making may restrict opportunities for detailed discussion. Nevertheless, a brief, non-judgemental and clinically focused history remains important for identifying the substances used, timing and routes of administration, relevant co-exposures, and immediate risks. EMS personnel should therefore balance the need for rapid assessment and stabilization with communication that facilitates disclosure without delaying time-critical care.

### 4.3 Slamsex and occupational safety considerations

A particularly high-risk form of chemsex is slamsex, defined as the intravenous administration of psychoactive substances during sexual activity. Slamsex is associated with increased risks of HIV transmission, hepatitis C infection, bacterial infections, overdose, and severe psychiatric complications (62). Slamsex may involve needle sharing and other unsafe injection practices (63). In prehospital settings, the presence of used or potentially contaminated sharps may pose an occupational risk of exposure to blood-borne pathogens for EMS personnel (64). Furthermore, prolonged sexual sessions involving multiple participants and polysubstance use may complicate scene management and increase the likelihood of unpredictable behavioral responses (62, 65). Consequently, EMS teams should maintain heightened awareness of occupational safety procedures, including the appropriate use of personal protective equipment, sharps management protocols, and post-exposure pathways when responding to suspected chemsex-related incidents. Beyond acute management, EMS encounters may also represent important opportunities for referral to harm reduction, addiction, sexual health, and community support services, which have been identified as key components of effective responses to chemsex-related harms (66). Facilitating access to these services may improve continuity of care and contribute to reducing the long-term harms associated with chemsex (66, 67).

## 5 Practical implications for EMS teams

Recognition of a chemsex context does not fundamentally change the established principles of prehospital management of acute intoxication. Initial assessment should continue to prioritize identification of the dominant clinical syndrome, stabilization of vital functions, and early recognition of life-threatening complications, particularly when the substances involved remain unknown. However, identifying the sexualized substance use context may provide additional clinically relevant information that informs targeted history taking, risk assessment, and subsequent management.

Given the frequent occurrence of polysubstance use, EMS teams should anticipate atypical and rapidly evolving presentations. Repeated clinical reassessment may be particularly important because patients can transition quickly between agitation, psychosis, sedation, respiratory depression, and cardiovascular instability. When a chemsex context is identified or suspected, targeted questioning should include repeated GHB/GBL dosing, concurrent stimulant and depressant use, and potential interactions between alkyl nitrites (poppers) and phosphodiesterase-5 (PDE5) inhibitors. Airway management, respiratory monitoring, temperature assessment, and continuous cardiovascular surveillance should therefore remain central priorities during patient evaluation and transport.

Operational factors are equally important. EMS teams should maintain awareness of scene safety issues, including behavioral disturbance, aggression, intravenous drug use, and the presence of contaminated sharps. Recognition of slamsex practices may help identify potential exposure to used injecting equipment and the need for appropriate sharps precautions. Establishing a non-judgemental communication environment may facilitate disclosure of clinically relevant information regarding substance use, sexual practices, and potential drug interactions. Questions about the sexual context of substance use should be clinically focused, respectful, and limited to information relevant to immediate patient care.

Finally, chemsex-related emergencies should be viewed not only as acute toxicological events but also as opportunities for engagement with broader health services. Where appropriate, referral pathways involving addiction services, sexual health clinics, mental health support, and harm reduction programs may contribute to improved long-term outcomes.

## 6 Future directions and research gaps

Despite growing recognition of chemsex as an emerging public health concern, significant gaps remain in the evidence base relevant to prehospital emergency care. Most published studies have focused on epidemiology, sexual health outcomes, HIV prevention, substance use patterns, and mental health consequences, whereas relatively little attention has been directed toward the challenges faced by EMS teams during chemsex-related emergencies. Current knowledge is largely derived from observational studies, emergency department cohorts, and community-based research. Most available evidence is retrospective and heterogeneous, with considerable variability in chemsex definitions, substances assessed, and reported outcomes, limiting direct applicability to prehospital care. Consequently, there is limited evidence regarding prehospital assessment strategies, optimal management approaches, operational demands, and patient outcomes following EMS encounters. To date, no prospective studies have specifically evaluated chemsex-related EMS activations or their impact on prehospital clinical decision-making and patient outcomes. In particular, the impact of chemsex-related emergencies on scene times, resource utilization, provider safety, and clinical decision-making remains poorly characterized. Additional research is needed to better understand the prevalence and characteristics of chemsex-associated EMS activations, identify predictors of severe clinical deterioration, and evaluate the effectiveness of existing prehospital management strategies. Development of evidence-based educational programs and clinical guidance tailored to EMS teams may further improve preparedness for these increasingly complex presentations. Future efforts should also explore opportunities to strengthen collaboration between EMS teams, emergency departments, addiction services, sexual health clinics, and community-based organizations. Such integrated approaches may improve continuity of care and support the management of both acute and long-term consequences of chemsex. The interpretation of findings presented in this review should also consider the limited availability of EMS-specific evidence, as many conclusions are necessarily informed by data derived from emergency department, toxicology, and community-based studies.

### 6.1 Study limitation

This narrative review has several limitations. First, the available evidence is heterogeneous and largely derived from observational studies, emergency department cohorts, toxicology reports, and community-based research. Second, EMS-specific data remain scarce, and many conclusions relevant to prehospital care are extrapolated from hospital-based studies. Also, variability in chemsex definitions, substances assessed, and reported outcomes across studies may limit direct comparisons and the generalizability of findings to different EMS systems and settings. Furthermore, the available evidence is predominantly derived from Western countries and urban settings, which may limit the global generalizability of the findings. Regional differences in legal frameworks, patterns of drug availability and use, and EMS organization, resources, and professional competencies may further influence the applicability of these findings across different healthcare systems. In addition, the narrative approach and non-systematic selection of sources may have introduced selection bias, potentially resulting in the omission or underrepresentation of relevant evidence. Finally, incomplete recognition and documentation of the chemsex context in routine emergency care records may lead to underestimation of chemsex-associated presentations and complicate the interpretation of temporal trends.

## 7 Conclusion

Chemsex-associated emergencies represent an emerging challenge for EMS teams and are frequently characterized by polysubstance exposure, rapidly evolving clinical presentations, and substantial operational complexity. A syndromic approach focused on recognition of hyperstimulatory, depressant, behavioral, and mixed toxidromic presentations may provide greater clinical utility than substance-specific assessment alone. Beyond acute intoxication, EMS teams must also address communication barriers, scene safety concerns, behavioral emergencies, and occupational risks associated with chemsex-related incidents. Although available studies document chemsex-associated emergency presentations, evidence regarding their frequency, temporal trends, and prehospital management remains limited. Further EMS-focused research is needed to support the development of evidence-based protocols, educational initiatives, and integrated care pathways for this increasingly complex patient population.
